# Anxiety, stress, and binge eating tendencies in adolescence: a prospective approach

**DOI:** 10.1186/s40337-021-00444-2

**Published:** 2021-08-03

**Authors:** Michele C. Lim, Sam Parsons, Alessia Goglio, Elaine Fox

**Affiliations:** Department of Experimental Psychology, University of Oxford, Anna Watts Building, Radcliffe Observatory Quarter, Woodstock Road, Oxford, OX2 6GG UK

**Keywords:** Eating disorder, Binge eating, Eating behaviour, Anxiety, Stress, Adolescence, Risk factor, Longitudinal, Structural equation modeling, RI-CLPM

## Abstract

**Background:**

Recent years have witnessed an increasing prevalence of binge eating tendencies in adolescence—warranting a clearer understanding of their underlying predisposing and precipitating factors. The current study investigated whether the interaction between high levels of anxiety and stress predicted increased levels of binge eating tendencies in a prospective cohort of adolescents (*N* = 324).

**Methods:**

Measurements were taken over three waves (*M* ages: 13.33, 14.48, 15.65) as part of the CogBIAS Longitudinal Study. Longitudinal associations between levels of anxiety and stress with binge eating tendencies were estimated using a random intercept cross-lagged panel model (RI-CLPM), which calculates within-person fluctuations over time while accounting for individual trait-like stability and between-person variations. Binge eating tendencies were measured by the Cognitive Restraint, Uncontrolled Eating, and Emotional Eating styles from the Three-Factor Eating Questionnaire-R18. Two models were created for each binge eating tendencies variable: (1) a basic model with anxiety and stress as independent variables; (2) an interaction model with an additional anxiety*stress interaction term. Model fit was assessed by SEM fit indices: *X*^*2*^, CFI, NFI, TLI, RMSEA, SRMR. Superior model fit was ascertained by a chi-square difference test (*p* < .05).

**Results:**

For Cognitive Restraint, the interaction model demonstrated superior fit to the data (*p* < .05). The anxiety*stress interaction at Waves 1 and 2 was significantly negatively associated with Cognitive Restraint at Waves 2 (*β* = −0.18, *p* = .002) and 3 (*β* = −0.14, *p* = .002)—suggesting that anxiety and stress interacted to predict increased binge eating tendencies linked with cognitive restraint over and above their independent effects. In contrast, the interaction term between anxiety*stress did not predict levels of Uncontrolled Eating or Emotional Eating over time.

**Conclusions:**

The results highlight the importance of increasing awareness of the interaction between concurrently high anxiety and stress as a potential risk factor for binge eating tendencies in young people.

**Trial registration:**

Not applicable.

## Plain English Summary

Binge eating tendencies have become an increasingly common phenomenon in adolescent populations. These behaviours involve consuming large amounts of food in a short period of time, during which one feels out of control and unable to stop. Episodes are often followed by marked emotional distress that can serve to perpetuate and maintain these tendencies. The current study examined a group of adolescents aged 13 to 16 over three testing periods spaced 12 to 18 months apart, to investigate whether high levels of anxiety and stress interacted to increase likelihood of binge eating tendencies in individuals over time. Results revealed that participants who experienced higher anxiety and stress than usual were more likely to score highly on binge eating tendencies measures, compared to when they experienced lower levels of anxiety and stress. Correspondingly, we recommend raising greater awareness in parents, educators, and health professionals of the link between high anxiety and stress and increased risk of binge eating tendencies, in order to facilitate better prevention, detection, and early intervention.

## Introduction

Recent years have seen a marked increase in binge eating tendencies in adolescent populations [1]. These behaviours involve consuming abnormally large amounts of food in a discrete period of time, during which one feels unable to stop [2]. Episodes are succeeded by marked emotional distress [2], while increased frequency is linked to impaired social functioning, anxiety and depression [3], and heightened risk for metabolic syndrome [4].

Binge eating tendencies present on a spectrum of severity within the general population—ranging from subclinical presentations of limited frequency to clinical binge eating disorder, with episodes occurring at least once a week for a minimum of three months [4]. In adolescent populations, binge eating disorder rates range from 1 to 5% [5], while subthreshold presentations of binge eating occur comparatively more commonly at rates ranging from 3.6 to 4.4% [6].

Adolescence poses a critical risk period for the development of binge eating tendencies [1], with studies identifying the first average age of onset at age 14 [7] and peak incidence at ages 16 to 17 [5]. During adolescence, puberty-induced physiological changes [8] and increased importance of interpersonal relationships can intensify preoccupations with one’s physical appearance [9]. Likewise, emerging identity development may facilitate adoption of a value system that equates self-worth with weight and shape [10]. Importantly, eating disturbances during adolescence are predictive of progression to clinical eating disorders in adulthood [11]—highlighting the need to better understand the contributing factors to binge eating tendencies in young people.

Dietary restraint theory [12, 13] posits that dieting shifts regulation of food consumption from physiological to cognitive control mechanisms—rendering one vulnerable to disinhibited eating when cognitive resources are depleted. This propensity is exacerbated by dichotomous ‘all-or-nothing’ thinking, which amplifies a seemingly minor lapse in one’s diet into a disinhibited eating spree or binge in vulnerable individuals [14]. Indeed, both dieting and dietary restraint are well-documented precedents of binge eating [15, 16], with one study citing an 18-fold increased risk of developing an eating disorder in 14-year-old girls who severely dieted [17].

Alternatively, escape theory [18] proposes that binge eating provides an ‘escape’, whereby the immediate act of consuming large amounts of food allows one to temporarily dissociate from experiences of negative affect. This theory is well illustrated in the robust links between binge eating and high levels of depression [19], anxiety [20], and stress [21]. Up to 65% of individuals with eating disorders report pre-morbid [22] and concurrent [23] anxiety that persist following recovery [24]. Likewise, adults with binge eating disorder endorse both high trait and state anxiety [25]. Importantly, this effect has been observed independently of general negative affect or depression [20, 26].

Critically, not all individuals with high anxiety turn towards binge eating [13]. Rather, concurrently high levels of stress may interact with anxiety to increase risk for binge eating tendencies [27]—particularly in those with strained relationships with eating or their weight and shape [28]. Indeed, binge eating typically emerges in the context of distress, panic, and catastrophic self-referential thinking [29, 30]—all of which manifest in anxiety symptoms and are exacerbated in stressful circumstances [31]. Likewise, individuals with binge eating disorder report higher same-day stress on days of binge eating episodes [21], whilst major stressful life events often precede the onset of bulimia nervosa and binge eating disorder [32]. In a similar vein, stressful circumstances appear to selectively increase overconsumption of hyperpalatable foods in individuals with higher trait anxiety [33].

Thus, stress and anxiety likely amplify each other’s influences on emotional coping strategies and eating behaviours to magnify risk towards binge eating in certain vulnerable individuals. This effect may be particularly salient in adolescence, where emotion regulation skills are still developing in the midst of interpersonal and social stressors [34].

To our knowledge, limited studies have investigated the interactive impact of anxiety and stress on binge eating tendencies in adolescents over a period of time [35]. Existing adult studies implicating anxiety and stress with disinhibited or binge eating have primarily relied on experimental inductions of stress in those with high trait anxiety [36] or ecological momentary assessment (EMA) methods in self-identified binge eaters [37]. However, these observations are based on acute effects over a period of hours or days, rather than months or years—leaving a gap in the literature regarding the temporal nature and magnitude of this relationship.

In a similar vein, much of the prospective research with younger participants has focused exclusively on depression [38, 39] or has examined anxiety independently rather than in conjunction with stress [8]—leading to a critical lack of understanding of their joint impact on binge eating tendencies [20].

Given the rising incidence of binge eating tendencies in adolescence [5] and the low remission rates from these behaviours [40], it is imperative to gain a more comprehensive picture of the contributing factors towards binge eating patterns to maximise prevention and early intervention.

The present study sought to address this gap by investigating whether the interaction between concurrently high levels of anxiety and stress was predictive of increased binge eating tendencies in adolescents from the ages of 13 to 16 via the CogBIAS Longitudinal Study dataset (CogBIAS-L-S [41, 42]). This interaction was operationalised by an anxiety*stress interaction term created by multiplying participants’ anxiety and stress scores.

Within this study, binge eating tendencies [43] were examined through the Cognitive Restraint, Uncontrolled Eating, and Emotional Eating subscales of the Three-Factor Eating Questionnaire-R18 (TFEQ-R18 [44]). Although not an explicit measure of binge eating, these subscales comprise cognitive and behavioural eating styles that have been associated with binge eating frequency and severity in community samples [45–47].

We used random intercept cross-lagged panel models (RI-CLPM [48]) to estimate whether individual participants who concurrently experienced more anxiety and stress than usual, consequently demonstrated higher indication of binge eating tendencies than usual on a within-person level.

Our main hypothesis was:

- The interaction between high levels of anxiety and stress would predict increased binge eating tendencies over and above their independent effects, as operationalised by an interaction term (anxiety*stress).

A secondary hypothesis was:

- Higher levels of depression, anxiety, and stress would independently predict increased binge eating tendencies.

## Methodology

### Participants

Data were selected from the CogBIAS Longitudinal Study (CogBIAS-L-S [41, 42]), which examined contributing factors to emotional and psychosocial resilience in adolescence. Data collection spanned four years with three testing waves spaced 12 to 18 months apart to optimise distinguishing between developmental stability and change. Ethical approval for the study was obtained from the National Health Service (NHS) National Research Ethics Service (NRES) Committee South Central (Project ID: 141833; 14/SC/0128). Participant mean ages across the three waves were 13.4 (*SD* = 0.7; *N* = 504; 55% female), 14.5 (*SD* = 0.6; *N* = 450; 56% female), and 15.7 (*SD* = 0.6; *N* = 411; 58% female). Exclusion criteria specified no existing neurological injuries or diagnosis of a psychiatric disorder.

The sample exhibited a low attrition rate of 18.5%—contrasting with the 26.5% average reported in a meta-analysis of similar studies (*N* = 143 [49]). Of the participants retained versus lost, an independent samples t-test revealed a main effect of gender, with greater retention of female participants, *t*(502) = −2.86, *p* = .004, *d* = 0.25. There was no effect of age, socioeconomic status, or ethnicity on participant dropout. Further details of the sample are available in the CogBIAS-L-S cohort paper [42].

We analysed data from participants who completed all three waves of testing. Due to missing data on one or more measures across multiple waves, 87 participants were removed. The final sample (*N* = 324; 67% female) was similar in composition to the original cohort, with mean ages of 13.33 (*SD* = 0.12), 14.48 (*SD* = 0.55), and 15.65 (*SD* = 0.53) across waves. Participants were predominantly Caucasian (74.69%), with a median socioeconomic status of “Bachelor’s degree” as the highest level of parental education (*Median* = 4, *IQR* = 2). Average participant body mass index (BMI; kg/m^2^) fell within the healthy range across all three waves (Wave 1: *M* = 19.89, *SD* = 3.27; Wave 2: *M* = 20.69, *SD* = 3.18; Wave 3: *M* = 21.24, *SD* = 3.20).

### Procedure

Participants were recruited from nine schools in South England. Parents or caregivers were sent a letter inviting their child to participate. The invitation disclosed the purpose of the study and ensured confidentiality and anonymity of their child’s data. Informed parental consent and adolescent assent were obtained for each testing session.

Within each testing session, participants were tested in a group setting at their respective schools, with sessions conducted during the school day. A controlled testing environment was simulated via induction of exam conditions (i.e., eyes on own screen, silence), with a teacher and two research assistants present throughout testing.

Measures were completed in a fixed order of six behavioural tasks and 13 self-report questionnaires covering mood, information processing biases, and eating-related attitudes and behaviours. Participants completed measures in one of two variations: one two-hour session on a single day or two one-hour sessions on separate days. Height (meters) and weight (kilograms) of each participant were measured privately on the day of testing in a separate room using a Seca portable height measure and Salter portable weight scales [42]. At each wave of testing, participants were paid £10 as compensation for their time.

All digital data was anonymised and stored in a password-protected server. Any personal identifying information and linkage codes were stored separately in a locked cabinet.

### Materials

Data from three self-report questionnaires were selected for these analyses. Continuous measures were chosen to reflect small but salient inter-individual differences and intra-individual growth that would otherwise be difficult to detect in categorically scored measures [50].

#### Binge eating tendencies: TFEQ-R18

Binge eating tendencies were assessed by the Three-Factor Eating Questionnaire-R18 (TFEQ-R18 [44])—an 18-item self-report evaluating three eating styles corresponding to distinct eating-related habits, attitudes, and behaviours. The measure is divided into three subscales: Cognitive Restraint (6 items; “I deliberately take small helpings as a means of controlling my weight”), Uncontrolled Eating (9 items; “Sometimes when I start eating, I just can’t seem to stop”), Emotional Eating (3 items; “When I feel blue, I often overeat”). Respondents rate how much each statement applies to them for 17 of the items on a 4-point Likert scale (1 = *Definitely false*, 2 = *Mostly false*, 3 = *Mostly true*, 4 = *Definitely true*) and one of the items on an 8-point Likert scale (1 = *No restraint in eating*, 8 = *Total restraint*).

Three subscale total scores are produced, with higher scores indicating greater endorsement of a given eating style [44]. Existing research has highlighted how eating styles captured by Cognitive Restraint [45], Uncontrolled Eating [46], and Emotional Eating [47] have been associated with general disordered eating tendencies [51] and increased risk of binge eating tendencies. Correspondingly, increased binge eating tendencies were operationalised by elevated levels of Cognitive Restraint [45], Uncontrolled Eating [46], and Emotional Eating [44].

While not a clinical measure for binge eating disorder, the TFEQ-R18 demonstrates sound criterion validity in differentiating between groups of individuals with disordered versus non-disordered eating as classified by the gold standard Eating Disorder Examination-Questionnaire (EDE-Q [52]). Likewise, it demonstrates acceptable criterion validity with the Compulsive Eating Scale (*r* = .65, *p* < .001)—a self-report measure assessing severity of binge eating disorder [53]. Within our study, Cognitive Restraint (ICC 2,*k* = .74), Uncontrolled Eating (ICC 2,*k* = .76), and Emotional Eating (ICC 2,*k* = .72) all demonstrated satisfactory test-retest reliability [54, 55].

#### Anxiety and depression: RCADS

Anxiety and depression were evaluated with the Revised Children Anxiety and Depression Scale (RCADS [56])—a 47-item self-report for ages 8 to 18. Respondents rate how much each statement applies to them based on a 4-point Likert scale (0 = *Never*, 1 = *Sometimes*, 2 = *Often*, 3 = *Always*). Item content is based on DSM-IV diagnostic criteria for anxiety disorders (e.g., “I worry bad things will happen to me”) and major depressive disorder (e.g., “I feel worthless”). The RCADS comprises six subscales: five corresponding to anxiety (generalised anxiety disorder, separation anxiety disorder, social phobia, panic disorder, obsessive-compulsive disorder); one corresponding to major depression.

We calculated a total anxiety (RCADS-A) score by summing responses across the five anxiety disorder subscales. A depression score was computed by summing scores across all 10 items of the major depressive disorder subscale, with higher scores indicating greater severity of symptoms. Within our study, both the anxiety (ICC 2,*k* = .82) and depression (ICC 2,*k* = .83) subscales demonstrated good test-retest reliability.

#### Stress: CASE

Stress was assessed by the Child and Adolescent Survey of Experiences (CASE [57])—a 38-item checklist of life events typical of ages 7 to 17. Life events of the CASE include parental divorce, changes in peer relationships, significant achievements, and school events. Respondents indicate whether a given life event has occurred in the past 12 months (*Yes* or *No*) then rate its perceived impact on a 6-point Likert scale (1 = *Really bad*, 2 = *Quite bad*, 3 = *A little bad*, 4 = *A little good*, 5 = *Quite good*, 6 = *Really good*). There is an option for respondents to include two additional significant life events that are rated in the same fashion.

The CASE provides two scores: a negative life events score (total number of items rated from 1 to 3) and a positive life events score (total number of items rated from 4 to 6). In the current study, stress was measured by the negative life events score and demonstrated satisfactory test-retest reliability (ICC 2,*k* = .74).

### Data analysis plan

To investigate the effect of the interaction between anxiety and stress on binge eating tendencies in participants over time, a random intercept cross-lagged panel model (RI-CLPM [48]) approach was employed using structural equation modeling (SEM [58]).

RI-CLPM extends traditional cross-lagged panel model (CLPM) approaches by separating between-person and within-person level stability and change over time through the inclusion of random intercepts [48]. This allows each participant to vary in their baseline and expected scores for each variable of interest across measurement points. Variance for each variable (e.g., anxiety) is divided into stable trait-like between-person individual differences (via random intercepts) and how much an individual deviates from their unique within-person baseline and expected scores for each variable at each measurement occasion (via a latent factor [59]).

These values are used by the model syntax to generate two key estimates of interest: (1) autoregressive parameters; (2) cross-lagged parameters. Autoregressive parameters denote the amount of within-person carry-over or the stability of an individual participant’s measurement on a given construct over time (e.g., amount of within-person carry-over of a participant’s levels of anxiety from Wave 1 to Wave 2). Cross-lagged parameters indicate the extent that an individual’s within-person deviation from their expected score on a given construct at one time point influences their within-person change on a different construct at a subsequent time point, after controlling for trait-like stability via random intercepts (e.g., how much one’s within-person deviation from their expected score in anxiety at Wave 1 is associated with within-person changes in their expected score for binge eating tendencies at Wave 2).

For the current study, RI-CLPM models were estimated using the *riclpmr* [60] and *lavaan* packages [61] with R statistical programming language (version 3.5.3 [62]) in RStudio (version 1.3.1073 [63]). The interactive impact of anxiety and stress was operationalised by centering the anxiety and stress variables and subsequently multiplying them to create an interaction term (anxiety*stress [64]).

**Fig. 1:**
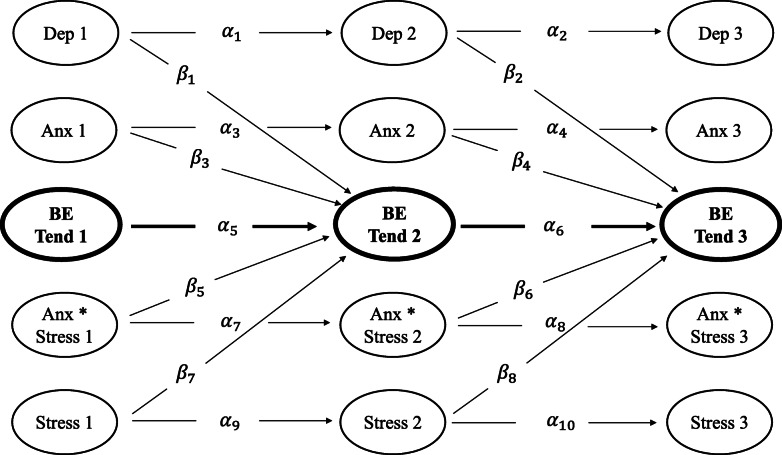
Schematic representation of an example interaction model with standardised autoregressive and cross-lagged parameters. All 10 autoregressive parameters are illustrated; only 8 of the 40 possible cross-lagged parameters are included for clarity. *Note.* α represents standardised estimates for latent autoregressive parameters. *β* represents standardised estimates for latent cross-lagged parameters. Numbers 1, 2, and 3 pertain to estimates of latent variable measurements at Waves 1, 2, and 3. ªDep – Depression. Anx – Anxiety. BE Tend – represents respective TFEQ-R18 eating styles indicative of binge eating tendencies (Cognitive Restraint, Uncontrolled Eating, Emotional Eating). Anx*Stress – Anxiety*Stress interaction. Stress – Stress

To evaluate whether the interaction between anxiety and stress predicted binge eating tendencies over and above their independent effects, a basic RI-CLPM and an interaction RI-CLPM were created for each of the three binge eating tendencies variables (Cognitive Restraint, Uncontrolled Eating, Emotional Eating) and subsequently compared for overall model fit and significance of cross-lagged parameters. In each basic model, depression, anxiety, and stress were included as independent predictors. In each interaction model, an additional anxiety and stress interaction term (anxiety*stress) was added (see Fig. 1 for an example model).

To maintain an equal number of terms across both models, we first created a respective interaction model for each binge eating tendencies variable that included depression, anxiety, and stress as independent predictors, with an additional anxiety by stress interaction term (anxiety*stress). A basic model for each binge eating tendencies variable was subsequently created by constraining any cross-lagged parameters including the anxiety*stress interaction term to zero. For clarity, we refer to the constrained interaction model as the ‘basic model’ throughout this paper.

Autoregressive paths were specified across each measurement interval (Wave 1 to Wave 2; Wave 2 to Wave 3) to capture stability between latent factors of the same variable (e.g., between anxiety at Wave 1 and anxiety at Wave 2). Cross-lagged paths were specified between time points for latent factors of all variables of interest (e.g., between anxiety*stress at Wave 1 and Cognitive Restraint at Wave 2 [59]).

The RI-CLPM approach accounts for stable between-person differences, therefore we did not include any stable covariates that may influence anxiety, stress, and binge eating tendencies (e.g., gender, socioeconomic status, BMI etc.) within the model specification [59]. We treated all variables as continuous in the models, as sum scores were used for each measure [65, 66]. All models were estimated using maximum likelihood (ML) estimation to account for nonnormality and nonindependence of data [58, 67].

All interaction (*N* = 324; *df* = 50) and basic (*N* = 324; *df* = 59) models for the binge eating tendencies variables were adequately powered to reject a misspecified model using the *semPower* package [68] in RStudio (version 1.3.1073 [63]). For the interaction models, an a priori power analysis noted that a sample size of *N* = 298 would yield approximately 90% power to reject a wrong model (*df* = 50) with an amount of misspecification corresponding to RMSEA = .05 with an alpha level of .05. For the basic models, an a priori power analysis noted that a sample size of *N* = 270 would yield approximately 90% power to reject a wrong model (*df* = 59) with an amount of misspecification corresponding to RMSEA = .05 with an alpha level of .05. As we only ran a total of six models, the main effect of interest would remain significant following even a conservative Bonferroni correction [69].

The following indices were used to assess each model’s goodness of fit to the data: (a) chi square (*X*^*2*^), (b) Comparative Fit Index (CFI), (c) Normed Fit Index (NFI), (d) Tucker Lewis Index (TLI), (e) Root Mean Squared Error of Approximation (RMSEA), (f) Square Root Mean Residual (SRMR). A significant chi square value (*p* < .05) indicated acceptable fit. CFI values > .95 [70], NFI values > .95, and TLI values > .90 all suggested good fit [71], while RMSEA and SRMR values < .05 indicated good fit [71].

To evaluate whether the interaction model demonstrated superior fit to the data, the following indices were employed: Akaike Information Criterion (AIC [72]), Bayesian Information Criterion (BIC [72]), and a chi-square (*X*^*2*^) difference test [73]. Smaller AIC and BIC values typically indicate superior model fit; however, both indices have been shown to inaccurately select overly complex (AIC) or overly parsimonious (BIC) models in sample sizes below 750 [72]. Due to the current study’s sample size (*N* = 324), the chi-square difference test was used as the primary indicator of superior fit [74]. A significant chi-square difference value (*p* < .05) would indicate that the ‘larger’ interaction model with the added effect of anxiety*stress is a better fit to the data than the ‘smaller’ basic model [74].

Finally, significance of latent cross-lagged parameters was determined by a threshold of *p* < .05 [75]. We hypothesised that the anxiety*stress interaction term would have a significant cross-lagged parameter with each binge eating tendencies variable (Cognitive Restraint, Uncontrolled Eating, Emotional Eating) at subsequent time points. In a similar vein, depression, anxiety, and stress were expected to show significant cross-lagged parameters with each binge eating tendencies variable at subsequent time points.

## Results

A total of six models were constructed: (1) Cognitive Restraint Basic, (2) Cognitive Restraint Interaction, (3) Uncontrolled Eating Basic, (4) Uncontrolled Eating Interaction, (5) Emotional Eating Basic, (6) Emotional Eating Interaction.

The main hypothesis that the interaction between anxiety and stress would predict increased binge eating tendencies over and above their independent effects was tested in three steps: (1) acceptable goodness of fit for the interaction model (via *X*^*2*^, CFI, NFI, TLI, RMSEA, SRMR); (2) superior fit of each interaction model to the current data compared to the basic model by a significant chi-square difference test (*p* < .05); (3) significant latent cross-lagged parameters between the anxiety*stress interaction term and each respective binge eating tendencies variable (Cognitive Restraint, Uncontrolled Eating, Emotional Eating) within each interaction model (*p* < .05). Evidence that a given binge eating tendencies variable was predicted by the interaction between anxiety*stress required all three criteria to be met.

Internal consistency for each measure was estimated using a criterion of > .70 with McDonald’s omega (*ω*), which demonstrates superior estimates to Cronbach’s alpha [76]. Table 1 presents descriptive statistics and internal consistency for each variable of interest.

**Table 1:** Descriptive statistics and internal consistency (McDonald’s ω) for variables across waves

	Wave 1 (*N* = 324)	Wave 2 (*N* = 324)	Wave 3 (*N* = 324)
Measure (possible range of scores)			
**RCADS anxiety (0–111)**			
Mean (*SD*)	13.91 (7.94)	14.76 (7.75)	14.22 (7.99)
*ω*	.89	.88	.89
**RCADS depression (0–30)**			
Mean (*SD*)	8.36 (5.62)	9.89 (6.22)	10.47 (6.45)
*ω*	.91	.91	.92
**CASE negative life events (0–40)**			
Mean (*SD*)	11.83 (8.86)	11.50 (8.98)	10.67 (7.99)
Anxiety*Stress			
Mean (*SD*)	34.24 (76.05)	33.33 (60.35)	36.83 (65.65)
**TFEQ-R18 Cognitive Restraint (6–24)**			
Mean (*SD*)	13.41 (4.21)	13.43 (4.51)	13.47 (4.74)
*ω*	.87	.92	*.*92
**TFEQ-R18 Uncontrolled Eating (9–36)**			
Mean (*SD*)	19.49 (5.83)	20.84 (5.72)	20.87 (5.64)
*ω*	.91	.89	.89
**TFE1-R18 Emotional Eating (3–12)**			
Mean (*SD*)	5.39 (2.53)	6.20 (2.84)	6.28 (2.82)
*ω*	.87	.89	.91

*Note*. *RCADS *Revised Children Anxiety and Depression Scale. *CASE* Child and Adolescent Survey of Experiences. Anxiety*Stress – anxiety*stress interaction term. *TFEQ-R18* Three-Factor Eating Questionnaire-R18. *ω* – McDonald’s Omega for internal consistency; > .70 indicates high internal consistency [76]

Fit measures for the basic and interaction models for each binge eating tendencies variable (Cognitive Restraint, Uncontrolled Eating, Emotional Eating) are found in Table 2. All values bar chi-square are reported to three significant digit decimal places in line with standard SEM practice [77].

**Table 2:** Basic and interaction model goodness of fit indices

	χ2	df	*p*	CFI	NFI	TLI	RMSEA [90% CI]	SRMR	AIC BIC
Cognitive Restraint									
Basic	89.48	59	.006**	.986	.961	.975	.040 [.022, .056]	.040	30,350 30,637
Interaction	68.65	50	.041*	.991	.970	.982	.034 [.097, .052]	.036	30,347 30,668
Uncontrolled Eating									
Basic	83.41	59	.020*	.989	.964	.980	.036 [.015, .052]	.038	30,808 31,095
Interaction	67.77	50	.048*	.992	.971	.983	.033 [.003, .052]	.034	30,810 31,132
Emotional Eating									
Basic	80.45	59	.033*	.990	.966	.983	.033 [.010, .051]	.036	29,337 29,625
Interaction	62.83	50	.105	.994	.973	.988	.028 [.000, .048]	.032	29,338 29,659

*Note:* χ2 – chi-square value, df – degrees of freedom, *CFI* Comparative Fit Index; > .95 indicates good fit. *NFI* Normed Fit Index; > .95 indicates good fit. *TLI* Tucker Lewis Index; > .90 indicates good fit. *RMSEA* Root Mean Squared Error of Approximation; < .05 indicates good fit. 90% CI – 90% confidence interval. *SRMR* Square Root Mean Residual; < .05 indicates good fit. *AIC *Akaike Information Criterion. *BIC* Bayesian Information Criterion

ªAll values bar chi-square are reported to three significant digit decimal places in line with standard SEM practice

*p < .05, **p < .01

In line with RI-CLPM practice, autoregressive and cross-lagged parameters of all models were constrained across waves to account for stable trait-like individual differences [48]. As a result, unstandardised coefficients (*B*) are equal across waves while standardised coefficients (*β)* may differ across waves [59]. Within our sample, standardised coefficients only varied slightly across waves.

Main output of standardised cross-lagged parameters in the interaction models and graphical representations of the significant parameters linked to each binge eating tendencies variable are available in the tables and figures below. Standardised coefficients for the parameters were reported to facilitate comparison across all variables of interest [78]. Mean structures and latent factors used to construct the autoregressive and cross-lagged parameters were omitted, and only coefficients from significant autoregressive parameters between the same variable and significant cross-lagged parameters with each binge eating tendencies variable were retained in the following tables and figures for clarity.

### Model comparison: independent versus interactive contributions of anxiety and stress in predicting binge eating tendencies

#### Cognitive restraint

The interaction model achieved excellent model fit to the data across all fit measures bar the chi-square value (see Table 2). As expected, a significant chi-square difference test, *X*^*2*^(9) = 20.83, *p* = .013, revealed that the interaction model demonstrated superior fit relative to the basic model.

**Fig. 2:**
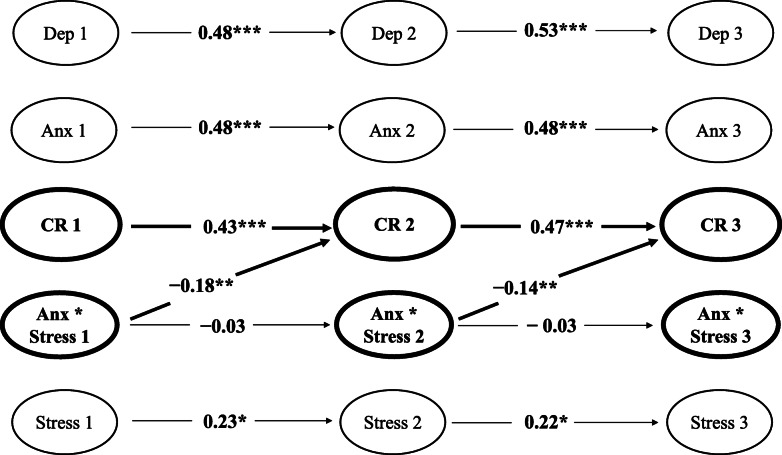
Cognitive Restraint interaction model with significant standardised estimates for autoregressive and cross-lagged parameters. *Note.* Values represent standardised estimates for significant latent autoregressive and cross-lagged parameters for Cognitive Restraint across Waves 1, 2, and 3. ªDep – Depression. Anx – Anxiety. CR – Cognitive Restraint. Anx*Stress – Anxiety*Stress Interaction. Stress – Stress. ^b^All autoregressive parameters are included. Only significant cross-lagged parameters and cross-lagged parameters pertaining to Cognitive Restraint are presented. All non-significant cross-lagged relationships are omitted for clarity. *p < .05, **p < .01, ***p < .001

In support of the main hypothesis, levels of the interaction between anxiety*stress at Waves 1 and 2 (see Table 3; Fig. 2) were negatively associated with the degree of Cognitive Restraint exhibited at Waves 2 (*β =* −0.18, *p* = .002) and 3 (*β* = −0.14, *p* = .002)—highlighting that anxiety and stress uniquely interacted to predict levels of binge eating tendencies operationalised by Cognitive Restraint in the current sample.

**Table 3:** Cognitive Restraint interaction model: cross-lagged parameters

Variable	*B* (*SE B*)	*β*	*z*	*p*	95% CI
Cognitive Restraint W2 predicted by:					
Depression W1	0.06 (.07)	0.07	0.85	.397	[−.08, .19]
Anxiety W1	0.02 (.05)	0.03	0.29	.772	[−.09, .12]
Stress W1	−0.04 (.04)	−0.07	−1.10	.269	[−.11, .03]
Cognitive Restraint W1	0.47 (.08)	0.43	5.74	< .001***	[.31, .64]
Anxiety*Stress W1	−0.10 (.03)	−0.18	−3.04	.002**	[−.16, −.04]
Cognitive Restraint W3 predicted by:					
Depression W2	0.06 (.07)	0.08	0.85	.397	[−.08, .19]
Anxiety W2	0.02 (.05)	0.03	0.29	.772	[−.09, .12]
Stress W2	−0.04 (.04)	−0.07	−1.10	.269	[−.11, .03]
Cognitive Restraint W2	0.47 (.08)	0.47	5.74	< .001***	[.31, .64]
Anxiety*Stress W2	−0.10 (.03)	−0.14	−3.04	.002**	[−.16, −.04]

Note. *B* – unstandardised latent estimate. *SE B* – standard error for unstandardised latent estimate. *β* – standardised latent estimate. *z* – z-value. 95% CI – 95% confidence interval

ªW1 – Wave 1. W2 – Wave 2. W3 – Wave 3

**p < .01, ***p < .001

Standardised cross-lagged parameters revealed no support for the secondary hypothesis of depression, anxiety, and stress independently predicting levels of Cognitive Restraint over time.

#### Uncontrolled eating

Good model fit was achieved in the interaction model across all fit measures (see Table 2). Contrary to what was expected, the interaction model did not demonstrate significantly better fit to the data than the basic model, *X*^*2*^(9) = 15.63, *p* = .08.

**Fig. 3:**
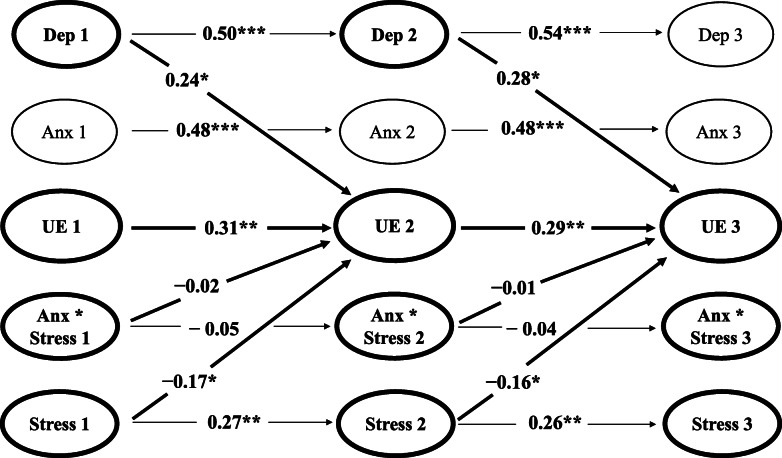
Uncontrolled Eating interaction model with significant standardised estimates for autoregressive and cross-lagged parameters. *Note.* Values represent standardised estimates for significant latent autoregressive and cross-lagged parameters for Uncontrolled Eating across Waves 1, 2, and 3. ªDep – Depression. Anx – Anxiety. UE – Uncontrolled Eating. Anx*Stress – Anxiety*Stress Interaction. Stress – Stress. ^b^All autoregressive parameters are included. Only significant cross-lagged parameters and cross-lagged parameters pertaining to Uncontrolled Eating are presented. All non-significant cross-lagged relationships are omitted for clarity. *p < .05, **p < .01, ***p < .001

Likewise, there was no support for the main hypothesis in the standardised cross-lagged parameters (see Table 4; Fig. 3). Levels of Uncontrolled Eating at Waves 2 and 3 were not significantly predicted by anxiety, stress, or the interaction between anxiety and stress at Waves 1 (*β =* −0.02, *p* = .804) and 2 (*β* = −0.01, *p* = .804).

**Table 4:** Uncontrolled Eating interaction model: cross-lagged parameters

Variable	*B* (*SE B*)	*β*	*z*	*p*	95% CI
Uncontrolled Eating W2 predicted by:					
Depression W1	0.21 (.09)	0.24	2.52	.010*	[.05, .38]
Anxiety W1	−0.03 (.07)	−0.05	−0.45	.652	[−.17, .11]
Stress W1	−0.11 (.05)	−0.17	−2.25	.024*	[−.20, −.01]
Uncontrolled Eating W1	0.29 (.09)	0.31	3.14	.002**	[.11, .46]
Anxiety*Stress W1	−0.01 (.04)	−0.02	−0.25	.804	[−.09, .07]
Uncontrolled Eating W3 predicted by:					
Depression W2	0.21 (.09)	0.28	2.52	.011*	[.05, .38]
Anxiety W2	−0.03 (.07)	−0.05	−0.45	.652	[−.17, .11]
Stress W2	−0.11 (.05)	−0.16	−2.25	.024*	[−.20, −.01]
Uncontrolled Eating W2	0.29 (.09)	0.29	3.14	.002**	[.11, .46]
Anxiety*Stress W2	−0.01 (.04)	−0.01	−0.25	.804	[−.09, .07]

Note. *B* – unstandardised latent estimate. *SE B* – standard error for unstandardised latent estimate. *β* – standardised latent estimate. *z* – z-value. 95% CI – 95% confidence interval

^a^W1 – Wave 1. W2 – Wave 2. W3 – Wave 3

*p < .05, **p < .01

The secondary hypothesis that depression, anxiety, and stress would independently predict binge eating tendencies was partially supported. Levels of depression at Waves 1 and 2 predicted levels of Uncontrolled Eating at Waves 2 (*β* = 0.24, *p* = .010) and 3 (*β* = 0.28, *p* = .011). Interestingly, levels of stress at Waves 1 and 2 were negatively predictive of Uncontrolled Eating at Waves 2 (*β =* −0.17, *p* = .024) and 3 (*β =* −0.16, *p* = .024).

#### Emotional eating

The interaction model demonstrated excellent fit across all fit measures bar the chi-square value (see Table 2). A significant chi-square difference test reflected that inclusion of anxiety*stress in the interaction model significantly improved fit to the data relative to the basic model in predicting levels of Emotional Eating, *X*^*2*^(9) = 17.62, *p* = .04.

**Fig. 4:**
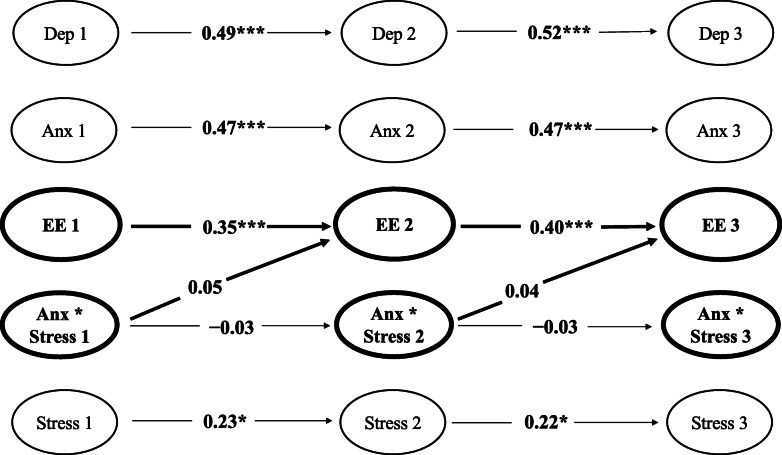
Emotional Eating interaction model with significant standardised estimates for autoregressive and cross-lagged parameters. *Note.* Values represent standardised estimates for significant latent autoregressive and cross-lagged parameters for Emotional Eating across Waves 1, 2, and 3. ªDep – Depression. Anx – Anxiety. EE – Emotional Eating. Anx*Stress – Anxiety*Stress Interaction. Stress – Stress. ^b^All autoregressive parameters are included. Only significant cross-lagged parameters and cross-lagged parameters pertaining to Emotional Eating are presented. All non-significant cross-lagged relationships are omitted for clarity. *p < .05, ***p < .001

However, the main hypothesis was ultimately not supported. Non-significant standardised cross-lagged parameters (see Table 5; Fig. 4) indicated that levels of the interaction between anxiety*stress at Waves 1 and 2 were not significantly associated with the degree of Emotional Eating exhibited at Waves 2 (*β* = 0.05, *p* = .387) and 3 (*β* = 0.04, *p* = .387).

**Table 5:** Emotional Eating interaction model: cross-lagged parameters

Variable	*B* (*SE B*)	*β*	*z*	*p*	95% CI
Emotional Eating W2 predicted by:					
Depression W1	0.05 (.04)	0.10	1.17	.244	[−.03, .13]
Anxiety W1	− 0.01 (.03)	− 0.02	− 0.21	.831	[−.07, .06]
Stress W1	−0.03 (.02)	−0.09	−1.32	.188	[−.07, .01]
Emotional Eating W1	0.41 (.08)	0.35	5.10	< .001***	[.25, .56]
Anxiety*Stress W1	0.02 (.02)	0.05	0.86	.387	[−.02, .06]
Emotional Eating W3 predicted by:					
Depression W2	0.05 (.04)	0.11	1.17	.244	[−.03, .13]
Anxiety W2	−0.01 (.03)	−0.02	−0.21	.831	[−.07, .06]
Stress W2	−0.03 (.02)	−0.08	−1.32	.188	[−.07, .01]
Emotional Eating W2	0.41 (.08)	0.40	5.10	< .001***	[.25, .56]
Anxiety*Stress W2	0.02 (.02)	0.04	0.86	.387	[−.02, .06]

Note. *B* – unstandardised latent estimate. *SE B* – standard error for unstandardised latent estimate. *β* – standardised latent estimate. *z* – z-value. 95% CI – 95% confidence interval

ªW1 – Wave 1. W2 – Wave 2. W3 – Wave 3

***p < .001

Likewise, contrary to our secondary hypothesis, levels of Emotional Eating at Waves 2 and 3 were not significantly predicted by levels of depression, anxiety, or stress at Waves 1 and 2.

## Discussion

The main hypothesis that the interactive impact of anxiety and stress would predict increased binge eating tendencies over and above their independent effects was partially supported. The interaction between anxiety and stress (anxiety*stress) was a strong negative predictor of Cognitive Restraint at subsequent waves within individual participants. Specifically, the two constructs were inversely related on a within-person level whereby: (1) higher levels of an interaction between anxiety and stress than usual at preceding time points predicted reduced Cognitive Restraint in individuals over time, and (2) lower levels of an interaction between anxiety and stress than usual predicted increased Cognitive Restraint in individuals over time.

The secondary hypothesis that depression, anxiety, and stress would each predict binge eating tendencies on a within-person level was also partially supported: participants who experienced higher levels of depression than usual exhibited more attitudes and tendencies of Uncontrolled Eating than usual at subsequent time points. Interestingly, Uncontrolled Eating was also associated with stress, whereby an increase in stress levels was linked to decreased levels of Uncontrolled Eating at subsequent waves.

### Interpretation of results for the main hypothesis: cognitive restraint

#### Interpretation 1: lower levels of the interaction between anxiety and stress and increased cognitive restraint are indicative of reduced binge eating tendencies

One interpretation of our results may be that concurrently lower levels of anxiety and stress facilitated greater Cognitive Restraint—consequently reducing likelihood of binge eating tendencies in participants [79, 80]. Cognitive Restraint in the TFEQ-R18 evaluates both the cognitive and behavioural extent to which one strictly regulates their food intake to monitor their weight. For example, “I do not eat some foods because they make me fat” comprises a regulatory behavioural component with a firm underlying psychological belief. Correspondingly, maintaining a consistently high level of Cognitive Restraint requires immense effortful control, behavioural regulation, and deliberate decision making that align with one’s goals of weight control [81]. As anxiety and stress have been shown to interfere with these capacities [82], it is plausible that concurrently reduced levels of both constructs may have optimised the behavioural execution of high Cognitive Restraint, resulting in reduced binge eating tendencies in the current sample.

#### Interpretation 2: higher combined levels of anxiety and stress and reduced cognitive restraint are indicative of increased binge eating tendencies

As previously mentioned, the disruptive impact of elevated anxiety and stress on cognitive control can interfere with the self-regulation capacities necessary for Cognitive Restraint (e.g., “I consciously hold back at meals in order not to gain weight” [82]). Given the proclivity of social, relational, and academic stressors in adolescence, it is possible that their accumulated impact interacted with high levels of anxiety to overwhelm participants’ already-taxed emotional and cognitive loads [83]. As a balanced emotional state and adequate cognitive capacity are fundamental to successful self-regulation [84], this may have led to momentary lapses into binge eating tendencies for certain individuals—highlighting how elevated levels of anxiety and stress may have reduced levels of restraint and increased likelihood of binge eating patterns in the current sample.

### Interpretation of results for the secondary hypothesis: uncontrolled eating

Our findings demonstrated that higher levels of depressive symptoms were associated with increased Uncontrolled Eating at subsequent waves [46]. Other prospective studies have noted similar effects of depression in predicting binge eating patterns in young adult women [19] and adolescents over a ten-year period [85]. Although levels of depression (*M* = 13.91–14.76; *SD* = 7.75–7.99) in the current sample were below clinically significant levels, subsyndromal depression has also shown robust links with binge eating tendencies [85, 86].

On a mechanistic level, depression is associated with reduced cognitive and affective flexibility [87], which are critical in effective emotional regulation [84]. Relatedly, deficiencies in emotional awareness and regulation have been associated with Uncontrolled Eating [88] and are particularly evident in adolescence where coping skills are still developing [89]. Thus, depressive symptoms may have possibly increased vulnerability to engaging in binge eating tendencies in the current sample as a means to cope with feelings of negative affect [88].

Contrary to our secondary hypothesis, our results demonstrated that stress levels were negatively predictive of Uncontrolled Eating: higher stress levels were linked to decreased levels of Uncontrolled Eating and thus, decreased indication of binge eating tendencies. Although elevated stress has been linked to bouts of disinhibited eating or overeating [90, 91], emotional stress can also induce loss of appetite and decreased caloric intake [92, 93]. In the context of our current sample, increased stress may have reduced participants’ appetites and desire for food—rendering them less susceptible to episodes of Uncontrolled Eating.

### Strengths, limitations, and future research

To our knowledge, the current study is one of the first to examine how the interaction between anxiety and stress may predict increased levels of binge eating tendencies on a within-person level. Past cross-sectional studies have observed that concurrently high anxiety and stress increase frequency of binge eating episodes in women with binge eating disorder [94], as well as women [95] and high school students [27] in the community. However, these relationships were examined individually, whereby anxiety and stress both simultaneously but separately increased incidence of binge eating. The current study contributes to the literature by highlighting that the two constructs combine to uniquely influence binge eating tendencies in an adolescent community sample.

While valuable in setting this study apart, employing a within-person RI-CLPM approach rather than a between-person CLPM analysis may have impacted our non-significant findings with Uncontrolled Eating and Emotional Eating. Analyses conducted with an RI-CLPM versus a CLPM approach have shown markedly different, and at times, opposing results despite using the same variables of interest and data [48]. For example, between-person studies have shown that, on average, individuals who experience higher anxiety and stress engage in more binge eating [27, 94]. However, it is possible that the opposite may occur on a within-person level [59]. Individuals who experience concurrently higher anxiety and stress *than usual* may actually temporarily lose their appetite, which could result in reduced rather than increased binge eating tendencies—demonstrating how effects can vastly differ amongst between-person and within-person level statistical comparisons. Thus, while a key strength, the novel RI-CLPM approach taken by this study may partially account for our failure to find significant relationships between the interaction of anxiety and stress with Uncontrolled and Emotional Eating.

In a similar vein, our use of self-report questionnaires may have impacted the accuracy of data provided due to self-report bias [96]. However, self-perceived attitudes and tendencies—albeit biased—are arguably central to the current study’s hypotheses, which are rooted in feelings of affect, attitudes, and subjective perceptions. Thus, while limited to a certain degree, the use of self-reports in this study is arguably a strength through capturing participants’ personal psychological experiences of anxiety and stress, and their resulting impact on binge eating tendencies.

With regards to measures, a key limitation lies in our use of the TFEQ-R18 Cognitive Restraint subscale to assess binge eating tendencies. While Cognitive Restraint itself has been associated with binge eating tendencies in the wider literature [97], existing research regarding its interpretation is somewhat inconclusive. Specifically, both high [45] and low [80] levels of Cognitive Restraint have been linked to increased binge eating tendencies. This discrepancy may stem from the existence of two distinct subtypes of Cognitive Restraint: (1) Rigid Control and (2) Flexible Control [98]. The former involves a tightly self-regulated approach to dietary intake that is implicated in disinhibited eating and higher BMI [99]. The latter denotes a more flexible approach to dieting and weight—exemplified by its links with stable eating patterns, lower BMI, and successful adherence to dietary and weight loss programs [100]. Within the current study, we chose to primarily interpret Cognitive Restraint as an expression of Flexible Control [98], whereby reduced Cognitive Restraint is associated with increased binge eating tendencies [79, 80, 98, 101]. However, due to the lack of consensus in the literature [45, 80], we caution this interpretation and suggest for further research to employ measures assessing both subdivisions of Rigid and Flexible Control to facilitate a clearer interpretation of Cognitive Restraint within samples.

Additionally, we suggest that future studies employ more detailed measures of eating attitudes and behaviours for clearer interpretation of participant data. Specifically, inclusion of additional restraint scales (i.e., Dutch Eating Behaviour Questionnaire [102]) would produce a more comprehensive picture of the relative frequency of adherence to regimented eating patterns versus binge eating episodes [103]. Likewise, inclusion of clinical assessment tools for binge eating (i.e., Binge Eating Scale [104]) and eating disorders (i.e., Eating Disorder Examination-Questionnaire [52]) would provide a better understanding of the severity and nature of binge eating behaviours and cognitions within participants.

### Implications

The results of this study highlight the need to raise greater awareness surrounding the link between anxiety and stress levels and elevated risk of disordered eating in early adolescence. Recommendations include increasing psychoeducation amongst parents, educators, and health professionals, whilst highlighting the importance of monitoring adolescents with moderate to high anxiety during stressful periods. This in turn would facilitate better prevention and early detection of binge eating tendencies that may emerge during this time.

## Conclusion

The current study both corroborates and extends existing research on the prospective relationship between anxiety and stress with indicators of binge eating tendencies. In addition to replicating the known independent links between depression and stress with binge eating tendencies, it is one of the first pieces of research to demonstrate how the interaction between levels of anxiety and stress is predictive of fluctuations in binge eating tendencies within individuals over an extended period of time. Specifically, adolescents who experienced concurrently higher anxiety and stress than usual were more likely to exhibit future binge eating tendencies—indicated by reduced levels of Cognitive Restraint. While further research is necessary to clarify the nuances of this relationship, this study highlights the interplay between anxiety and stress as a likely contributor to increased binge eating tendencies in young individuals.

## Data Availability

The data and code used for the analyses within this study can be found online: https://osf.io/8b2q7/?view_only=353e9e2a8474475faead8fb529f29b91.

## Abbreviation

RI-CLPM: random intercept cross-lagged panel model
